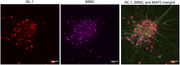# Investigating the interaction of APOE4 with TDP43 mislocalisation in driving neurodegeneration

**DOI:** 10.1002/alz70855_099312

**Published:** 2025-12-23

**Authors:** Antonio Rocco Fusciardi, Jonathan Mill, Akshay Bhinge

**Affiliations:** ^1^ University of Exeter, Exeter, Devon, United Kingdom

## Abstract

**Background:**

APOE4 is one of the strongest genetic risk factors for developing Alzheimer's disease. Additionally in 80% of severe cases of Alzheimer's disease, TDP43 inclusions have been found in post mortem brain slices, this suggests TDP43 inclusions may play a role in the progression of Alzheimer's disease. This project aims to investigate if a dual‐hit model of both APOE4 expression and TDP43 mislocalisation can result in a synergistic increase in neurodegeneration in IPSC‐derived cortical neurones.

**Method:**

We optimised a technique for generating BRN2 positive cortical neurones from human IPSCs via over‐expression of various transcription factors via lentiviral vectors and addition of various small molecules. Immunostaining and RTQPCR techniques were used to identify these population of neurones. We intend to create our dual hit APOE4‐TDP43 mislocalisation model via overexpression of APOE4 via lentiviral factors. TDP43 mislocalisation will be achieved by use of TDP43‐GFP tagged IPSCs, and the expression of GFP‐specific nanobodies tagged with a nuclear export signal. Quantification of Alzheimer's disease biomarkers such as amyloid beta and phospho‐tau will be performed via indirect sandwich ELISA and western blotting. Neurodegeneration will be quantified via multiplex cell viability assay utilising Calcein AM and ethidium homodimer‐1. RNA sequencing will identify differences between our dual hit model and controls. We will examine epigenetic changes between our model and compare to Alzheimer's disease to assess the model's validity.

**Results:**

Furthermore, we are generating protocols to produce cortical neurones for both cortical layers 2‐3 and deeper layers positive for markers such as ISL‐1, and CTIP2. Our lab has already previously generated GFP‐specific nanobodies that can be expressed via lentiviruses, in addition to a GFP tagged TDP43 IPSC line. We are currently generating lentiviral vectors to transduce our cells with APOE4.

**Conclusion:**

Overall our results show great promise as several previous studies provided sufficient justification that demonstrates a role for TDP43 mislocalisation in Alzheimer's disease, thus the establishment of a synergistic effect on the progression of neurodegeneration could open new pathways for research and clinical studies.